# Supplementary material for: Chromothripsis during telomere crisis is independent of NHEJ, and consistent with a replicative origin
Source: Genome Res. 2019 May;29(5):737–49. doi: 10.1101/gr.240705.118 (PMC6499312; doi:10.1101/gr.240705.118)
Supplement: Supplemental Material [file supp_gr.240705.118_Supplemental_file_1.zip › contigs/annotated_contigs/DB69/contig.2.DB69_length_277_mean_cov_9.68592057762.docx]

**DB69_length_277_mean_cov_9.68592057762**

```
TTTTTCTTCATTTCATAATTCATACTAAAAATAATTTTGTCGTGTAAAGAAATGAGTGTTAAAAAATTATCCACTCCAGG|TGT|GGCT
 >chr1:106669266-106669349 - E=1e-38 p=3e-02                                     >chr1:
TCCCCTCTTATATGCTTACCCTATTGGCATTAACCTCAGCAAAGCAGTCAATATATTTAATATTGTCAGATAGTATATATATGAATTTT
106668550-106668747 - E=1e-107
TCTTTTCAAACATTCTTATTGTTCTCTATTTTCTTCAGAGTACAAGCCATGGCCCTAAACCTACAATGGCCTATGGGTTCCTGGTATGT

TTCCAGTTCATT
```
